# A systems biology approach using metabolomic data reveals genes and pathways interacting to modulate divergent growth in cattle

**DOI:** 10.1186/1471-2164-14-798

**Published:** 2013-11-18

**Authors:** Philipp Widmann, Antonio Reverter, Marina R S Fortes, Rosemarie Weikard, Karsten Suhre, Harald Hammon, Elke Albrecht, Christa Kuehn

**Affiliations:** 1 Leibniz Institute for Farm Animal Biology, Institute for Genome Biology, Genome Physiology Unit, Dummerstorf, Germany; 2 Leibniz Institute for Farm Animal Biology, Institute for Nutritional Physiology “Oskar Kellner”, Dummerstorf, Germany; 3 Leibniz Institute for Farm Animal Biology, Institute for Muscle Biology and Growth, Dummerstorf, Germany; 4 CSIRO Animal, Food and Health Sciences, Brisbane, Australia; 5 Queensland Alliance for Agriculture and Food Innovation, The University of Queensland, Gatton Campus, Gatton, Australia; 6 Department of Physiology and Biophysics, Weill Cornell Medical College in Qatar, Education City, Qatar Foundation, P.O. BOX 24144, Doha, State of Qatar; 7 Institute of Bioinformatics and Systems Biology, Helmholtz Zentrum München, German Research Center for Environmental Health, Neuherberg, Germany

**Keywords:** Cattle, SEGFAM, Systems biology, Metabolomics, Genome-wide association study, Divergent growth, Puberty

## Abstract

**Background:**

Systems biology enables the identification of gene networks that modulate complex traits. Comprehensive metabolomic analyses provide innovative phenotypes that are intermediate between the initiator of genetic variability, the genome, and raw phenotypes that are influenced by a large number of environmental effects. The present study combines two concepts, systems biology and metabolic analyses, in an approach without prior functional hypothesis in order to dissect genes and molecular pathways that modulate differential growth at the onset of puberty in male cattle. Furthermore, this integrative strategy was applied to specifically explore distinctive gene interactions of non-SMC condensin I complex, subunit G (*NCAPG*) and myostatin (*GDF8*), known modulators of pre- and postnatal growth that are only partially understood for their molecular pathways affecting differential body weight.

**Results:**

Our study successfully established gene networks and interacting partners affecting growth at the onset of puberty in cattle. We demonstrated the biological relevance of the created networks by comparison to randomly created networks. Our data showed that GnRH (Gonadotropin-releasing hormone) signaling is associated with divergent growth at the onset of puberty and revealed two highly connected hubs, *BTC* and *DGKH*, within the network. Both genes are known to directly interact with the GnRH signaling pathway. Furthermore, a gene interaction network for *NCAPG* containing 14 densely connected genes revealed novel information concerning the functional role of *NCAPG* in divergent growth.

**Conclusions:**

Merging both concepts, systems biology and metabolomic analyses, successfully yielded new insights into gene networks and interacting partners affecting growth at the onset of puberty in cattle. Genetic modulation in GnRH signaling was identified as key modifier of differential cattle growth at the onset of puberty. In addition, the benefit of our innovative concept without prior functional hypothesis was demonstrated by data suggesting that *NCAPG* might contribute to vascular smooth muscle contraction by indirect effects on the NO pathway via modulation of arginine metabolism. Our study shows for the first time in cattle that integration of genetic, physiological and metabolomics data in a systems biology approach will enable (or contribute to) an improved understanding of metabolic and gene networks and genotype-phenotype relationships.

## Background

Body growth is a key trait in livestock production, and growth rate is an important predictor for the improvement of meat production efficiency in cattle. Recent studies successfully identified polymorphisms with major impact on growth related phenotypes in many species including cattle. For example, polymorphisms in pleiomorphic adenoma gene 1 (*PLAG1*) [1-3], non-SMC condensin I complex, subunit G (*NCAPG*) [4-6], and myostatin (*GDF8*, also known as *MSTN*) [7,8] were found to exert major effects on stature, postnatal growth and muscle development, respectively. Interestingly, several of these loci seem to be conserved modulators of mammalian growth, because concordant growth associated polymorphisms were detected in several species [7,9-18]. However, it is unclear which genes and pathways interact to regulate differential growth. One main reason for this is that growth as a complex phenotype is controlled by a large number of genes and environmental factors. Additionally, epigenetic and pleiotropic mechanisms as well as a multitude of small-effect genes make the detection of contributing polymorphisms challenging [19].

Recently, studies combining genetic with metabolomic data were very successful in detecting genes and pathways that are implicated in the manifestation of complex traits [20-22]. Instead of restricting genome-wide association studies (GWAS) on the complex target trait itself, GWAS in those studies also considered quantitatively measured metabolites from a comprehensive metabolomic analysis. This approach yielded novel insights in the physiological pathways that are important for the manifestation of the complex trait of interest. Metabolites are often intermediate phenotypes that represent genetically determined links between the genome and an animals’ physiological status or a complex trait. Thus, GWAS on metabolites are often more powerful for identifying associations between genes involved in metabolite conversions and complex traits due to the larger effect sizes obtained after regression of polymorphisms in those genes on metabolites than on a complex trait [20]. In conclusion, quantitatively determined metabolites turned out to be highly valuable in detecting genes and pathways that are involved in the manifestation of complex traits.

In addition to the use of metabolomics data, the application of systems biology methods is another approach for improving the analysis of the background of complex traits. Studies conducted by Fortes *et al*. [23,24] recently showed that systems biology approaches are powerful tools for the dissection of complex traits. Systems biology aims at obtaining a comprehensive view about the structures and dynamics within a system by using data from different levels of knowledge (e.g., genomics, transcriptomics, metabolomics, proteomics) [25]. Fortes *et al*. [23,24] applied a novel systems biology approach in order to infer a network, based on additive gene effects, which assembles interactions between functionally relevant genes for the trait of interest. Their approach turned out to be easily accessible, because it used data from common GWAS and merged data from different levels of information to describe the complex trait of interest in a comprehensive way.

Both concepts, exploiting metabolomic data or applying systems biology, contributed individually to the dissection of complex traits. The present study now combined both approaches for examining the physiological background of genetically determined divergent growth at the onset of puberty in cattle. For this purpose, we used a unique resource population (SEGFAM) which had been established for the examination of the background for divergent muscle protein accretion in cattle [26]. In this resource population, the onset of puberty was the main interval for differential growth [6]. Weikard *et al*. [6] identified mutations in the *NCAPG* and *GDF8* gene associated with divergent growth at puberty and revealed interacting metabolites and physiological pathways at this time point. Based on these results, the present study aims to generate a gene-gene interaction network for genetically divergent growth at the onset of puberty and to dissect the resulting network regarding physiological pathways. A further target of the study was the analysis of regulatory effects of *NCAPG* and *GDF8* on growth at the onset of puberty. To our knowledge, this is the first study combining metabolomic data with a systems biology approach in order to examine cattle growth.

## Methods

### Animals and sample collection

This study included 152 male F_2_ individuals from a Charolais x German Holstein resource population (SEGFAM) [26]. The animals were generated by multiple ovulation and embryo transfer and were kept under standardized feeding and husbandry conditions in the experimental unit of the Leibniz Institute for Farm Animal Biology (FBN) in Dummerstorf, Germany. Feeding and housing conditions were as described earlier [6]. Essentially, immediately after birth, the calves were removed from the mother and fed a milk replacer diet. After weaning at day 121, the animals were fed a diet consisting of hay and concentrate *ad libitum*. The hay to concentrate ratio was 1:3, and the energy content of the concentrate was 11.3 MJ ME/kg dry matter. At the age of 574 days, the animals were slaughtered in the slaughter house of the FBN. For the metabolomic analyses, blood samples were collected at 240 days of age with a standardized protocol. All samples were taken at 7:30 AM after a fasting period of 12 hours. The blood samples were collected in EDTA containing tubes (Sarstedt AG & Co, Nümbrecht, Germany) and immediately stored on ice to interrupt further processing of metabolites and enzyme activities. Within 30 min, blood samples were taken into the laboratory of the FBN where plasma was obtained after blood centrifugation. Plasma samples were stored at −80°C until they were used for the metabolomic analyses. For DNA genotyping, blood samples were obtained at slaughter and leucocytes were extracted and stored at −20°C until DNA isolation.

All experimental procedures were carried out according to the German animal care guidelines and were approved and supervised by the relevant authorities of the State Mecklenburg-Vorpommern, Germany.

### Phenotypes

Total body weights (tw) and daily weight gains (dwg) were determined from monthly weight recording of the animals. In our analyses, total weights were investigated for the following time points: 0 (birth), 4, 6, 9, 12, 15 and 18 months of age. Based on these measurements, average daily gains for the following time spans were calculated: 0–18, 4–18, 4–6, 6–9, 9–12, 12–15, and 15–18 months.

A total of 221 known metabolites were quantified from serum samples of 152 individuals with the help of electrospray ionization tandem mass spectrometry (ESI-MS/MS), using the Biocrates targeted metabolomics technology [6,27]. This procedure led to a target-oriented quantification of metabolites from the following substance classes: acylcarnitines, amino acids, hexoses, glycerophospho- and sphingolipids. Internal standards guaranteed standardized measurements. In summary, 48 acylcarnitines [free carnitine (C0), acylcarnitines (Cx:y), hydroxyacylcarnitines [C(OH)x:y] and dicarboxyacylcarnitines (Cx:yDC)], 18 amino acids, 9 lysophosphatidylcholines (lyso_PC_Cx:y), 70 phosphatidylcholines [diacylglycerophosphatidylcholines (PC_aa_Cx:y), acyletylglycerophosphatidylcholines (PC_ae_Cx:y), 16 sphingomyelins [sphingomyelins (SM_Cx:y), N-hydroxyldicarboacylacyloylsphingosyl-phosphocholines [SM(OHCOOH)x:y] and N-hydroxylacyloylsphingosyl-phosphocholines [SM(OH)x:y], 8 biogenic amines and 52 sugars were quantified.

### Genotyping and quality control

The 152 F_2_ animals were genotyped with *Illumina® Bovine SNP50 v2* chips. The chips were processed according to the *Illumina® Infinium HD Assay Ultra* guidelines and read out on an *Illumina® iScan* system. Quality control was carried out using *Illumina® Genome Studio v2011*. In order to increase data quality, clusters for all SNPs with either a call frequency < 0.98, a GenTrain Score < 0.68 or a Chi^2^-test for deviation from Hardy-Weinberg equilibrium < 0.005 were checked and manually re-clustered, if possible. After manual re-clustering, only autosomal SNPs with a call frequency > 0.85 and a minor allele frequency > 0.01 as well as all samples with a call rate > 0.98 were included in further analyses.

### Data analysis

#### GWAS

In a first step, GWAS were carried out for total body weight traits (n = 7), daily weight gain traits (n = 7) and all metabolomic traits (n = 221). The additive effects for each SNP on each trait were calculated using *Qxpak v5.05* software [28] fitting the following mixed model:

(1) $$y_{i}=X_{i}\beta +Z_{\mathrm{ik}}g_{k}+u_{i}+e_{\mathrm{ik}}$$

where *y*_*i*_ contains the phenotypic records of animal *i*, *X*_*i*_ is the *i*th row of an incidence matrix, *β* contains the fixed effects to be estimated, *Z*_*ik*_ represents the genotype of animal *i* at locus *k* and takes on the values of 1, 0, or −1, *g*_*k*_ contains the additive effect of locus *k*, *u*_*i*_ is the infinitesimal polygenic effect of animal *i* as estimated by Qxpak via a pedigree based additive animal model, and *e*_*ik*_ is the residual variance, with random effects distributed as multivariate normal with mean equal to 0 and covariance equal to:

$$\mathrm{Cov}\left(\begin{array}{l} g_{k}\\ u\\ e \end{array}\right)=\left(\begin{array}{ccc} \sigma_{g}^{2} & 0 & 0\\ 0 & U & 0\\ 0 & 0 & R \end{array}\right)$$

Different fixed effects for the metabolomic traits and the weight traits were fitted in model (1): for the metabolomic traits, the year of sampling and day of the metabolomic measurements were included; whereas for the weight traits the year of birth was considered. Statistical significance (p-values) for each SNP-trait combination was determined by *Qxpak* via a likelihood ratio test.

### Association weight matrix and partial correlation information theory

In order to exploit the resulting GWAS data beyond single-trait-single-SNP analyses, the *Qxpak* output was processed using a network approach already described by Fortes *et al.*[23,24]. This approach assumes that genes with strongly correlated additive effects on a complex trait are likely to share genetic regulation acting on the expression of the respective trait. Developing the gene-gene interaction network requires a set of genes with an initial experimental indication of effects on the complex target trait (in this study the results from the GWAS for growth at onset of puberty) and the significant interactions between these genes. Merging GWAS results and positional genomic information of SNPs, we assembled the respective set of potentially effective genes applying an association weight matrix (AWM) approach [23,29]. Phenotypes in the AWM approach are subdivided into key phenotypes and supportive phenotypes. Key phenotypes (e.g., total weight and daily weight gain) are the primary focus when assembling the AWM, because they are of highest physiological relevance for the complex trait (e.g., growth at onset of puberty). Supportive phenotypes (e.g., metabolites) are parameters that can be assumed to have some functional relationship with the key phenotypes. Adding the supportive phenotypes enriches the AWM with further biological information about the complex trait. As AWM works best with data from 10–20 different (as independent as possible) phenotypes [29], the initial data set had to be reduced. For the present study focusing on divergent growth at the onset puberty, total weight at month 9 (tw273) and daily weight gain from month 6 to 9 (dwg273) were selected as key phenotypes for three reasons: (i) the targeted time interval specifically corresponds to the onset of puberty in cattle [6], (ii) in the targeted time interval, the *NCPAG* and *GDF8* genes that influence growth and muscle traits in a variety of species [6,7,17,30] displayed two major loci with strong divergent effects on postnatal growth in our resource population [6], and (iii) the metabolomic data obtained at d240 was relevant because this time point for measurement matched the growth period of most interest.

**Figure 1:**
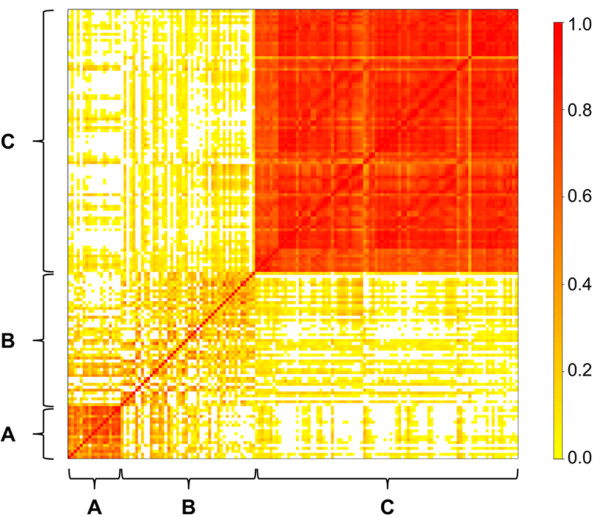
**Heat map of metabolites.** Each square in the heat map represents the spearman correlation coefficient between two metabolites (each column or row, respectively, represents a distinct metabolite). The strength of correlation is visualized with a color gradient ranging from white (no correlation) over yellow (little correlation) to red (high correlation). **(A)** 18 amino acids, **(B)** 46 acylcarnitines (including carnitine), **(C)** 8 lysophosphatidylcholines, 66 phosphatidylcholines and 16 sphingomyelins.

The selection of supportive phenotypes from the total set of metabolites aimed at low data redundancy in order to add extensive and diverse information to the AWM. From a correlation analysis, it was evident that the amino acids as well as the lysophosphatidylcholines, phosphatidylcholines and sphingomyelins form two highly homogenous groups, in which serum metabolite levels are strongly correlated (Figure 1). Thus, there is a high degree of redundancy within these two metabolite groups, and serum metabolite levels are highly predictive within these groups. On the other hand, the acylcarnitines form a very heterogeneous sub-group (according to their correlations) containing less redundant information (Figure 1). As the AWM greatly benefits from a set of supportive phenotypes with little redundancy (highly correlated data would only contribute redundant information to the AWM) [29], we preferentially selected metabolites from the heterogeneous group of acylcarnitines for the final set of supportive phenotypes in the AWM. Prioritizing low redundancy and high potential biological relevance of the metabolites for the complex trait, the final supportive phenotype set comprised the amino acids arginine and lysine, the acylcarnitines C0, C2 C5, C8:1, C14 and C18, the phosphatidylcholines PC_aa_C32:0 and PC_ae_C36:1 and the sphingomyeline SMC_20:2. These metabolites are involved in growth related processes like energy metabolism (amino acids, acylcarnitines), fatty acid trafficking and lipid metabolism (acylcarnitines, phosphatidylcholines, sphingomyelins) as well as signal transduction (phosphatidylcholines, sphingomyelins) and are therefore biologically relevant for the complex trait growth.

After generating the GWAS and the selection of key and supportive phenotypes, the AWM was built essentially as described [23,29]. Briefly, the AWM approach requires two tables as input. Both tables contain row wise the SNPs that passed the quality control and column wise the examined phenotypes. The cells of the first table are filled with the association significance (p-values) between SNPs and each phenotype, whereas cells in the second table contain the additive effects of each SNP on each phenotype. The p-values and additive effects of SNPs in the *NCAPG* and *GDF8* genes (*NCAPG I442M* and *GDF8 Q204X*), which are known for their substantial effects on postnatal growth in the SEGFAM population [4,6], had to be manually added to these tables, because the bovine 50k SNP chip did not harbor any SNP within a 2500 bp distance to *NCAPG* and *GDF8*. For this purpose the p-values and additive genetic effects for *NCAPG I442M* and *GDF8 Q204X* were calculated separately for each phenotype with Qxpak v5.05 applying model (1). Subsequently, all additive effects were normalized column wise in order to allow comparisons across traits. SNPs that were associated to any of the two key phenotypes at a threshold of p ≤ 0.05 were added to the AWM. In the next step, the average number of supportive phenotypes to which these SNPs were associated at p ≤ 0.05 was determined. This average was set to A_P_. Subsequently, all SNPs that were associated to at least A_p_ supportive phenotypes were added to the AWM. From the resulting set of SNPs, all SNPs that were more than 2500 bp away from the closest gene were then discarded ensuring that the final AWM only contains SNPs that are either located in a gene, a promoter or near a promoter region. For this purpose, all SNPs were mapped against the UMD3.1 assembly (ftp://ftp.cbcb.umd.edu/pub/data/assembly/Bos_taurus/Bos_taurus_UMD_3.1/, accessed: 06/20/2013). Finally, in case of multiple SNPs per gene, only the SNP with the highest number of associations to phenotypes and the lowest average p-value was kept to obtain a “one gene – one SNP” relationship. After SNP/gene selection, the AWM was built up by assigning the standardized additive effect of the *i*th SNP on the *j*th phenotype to each {*i*,*j*} cell of the AWM matrix, whereas the SNP symbols were replaced by the official gene symbols determined as indicated above.

Correlations among phenotypes (columns) and gene/SNP effects (rows) in the AWM were visualized and analyzed with the PermutMatrix 1.9.3 software [31]. The AWM contains SNPs representing genes, which were selected at a quite relaxed statistical threshold of p ≤ 0.05 without any correction for multiple testing. To account for this, the Partial Correlation coefficient Information Theory (PCIT) approach [32] was applied to determine data driven statistical significance thresholds for gene-gene interactions within the AWM. PCIT combines partial correlations (PC) with information theory (IT) and creates the gene-gene interaction network. In the first step, PCIT determines PCs for all possible trios of genes in the AWM, with the PC between genes A and B given gene C indicating the strength of the linear relationship between A and B that is independent of C. In the second step, PCIT compares the PCs between two genes relative to the PCs between each of these two genes and any other gene in the AWM in order to determine thresholds for significant gene-gene interactions. This step makes PCIT appealing for threshold determinations in co-association networks, because thresholds for significant gene-gene interactions are determined from the data itself. Since PCIT creates a very complex dataset of gene-gene interactions, the present study focuses on significant connections (according to PCIT) with a |PC| ≥ 0.80. This subset of data represents an acceptable balance between the number of significant interactions and the amount of data that could efficiently be analyzed with the visualization software Cytoscape [33].

It has to be pointed out that, although PCIT gives information about the direction of the partial correlations (positive or negative) between two genes, this information was ignored when constructing the gene-gene interaction network.

### Network analysis

Cytoscape [33] was applied for the analysis and visualization of the resulting network from PCIT in which nodes represent genes presumably relevant for growth at onset of puberty and edges represent significant partial correlations between additive gene effects, as determined by PCIT. In order to test if this network did not just represent a random accumulation of gene-gene interactions, 10 random networks were built and compared with the original growth network. Each of the random networks was created independently by applying the following procedure: At first, the standardized additive effects in the previously established AWM were column wise randomized. This procedure generated a random AWM, comprising SNP-trait associations completely independent from the associations obtained by the original GWAS results. The randomized AWM was then used as input for the PCIT approach in order to identify genes with significant partial correlations of additive effects. Due to the random nature of the AWM, these partial correlations could only arise by chance. The PCIT output was subsequently used as input for Cytoscape [33] in order to visualize the topology of the random network and to determine the number of connections per gene in the network. Finally, the average number of connections per gene across all 10 independent random networks was calculated. If the growth network indeed displayed gene-gene interactions relevant for growth, then the random networks were expected to include fewer genes and a lower number of significant gene-gene interactions than the growth network.

In order to evaluate how much of the information in the growth network was contributed by including metabolomic data, we created a network that solely based on the metabolomic trait data. We then analyzed the amount of overlap of this network with the full growth network generated from physiological and metabolomic data. The metabolic network was created as follows: After removing the columns containing the p-values and additive effects of the two weight traits (total weight at month 9 and the daily weight gain from month 6 to 9) from the two previously described AWM input tables, an AWM was generated by normalizing the additive SNP effects and by selecting all SNPs that were associated to more than A_P_ metabolites (A_P_ value taken from the initial analysis, see above). The subsequent steps for AWM and PCIT network generation and visualization were conducted as described above. Applying the Cytoscape option “Network merge”, the genes and interactions that overlapped between the full growth network and the metabolomic data network were determined. This overlap was considered as the amount of information that was at least contributed by the metabolomic trait data.

In order to test if certain Gene Ontology terms [34] (http://www.geneontology.org/, accessed: 04/12/2013) were significantly overrepresented in the growth network, GO term enrichment analyses for 241 biological processes, 51 cellular components and 206 molecular functions were carried out on the network using PANTHER 8.0 [35,36]. To this end, GO term enrichment was determined comparing all genes from the growth network with the reference list containing all genes from the UMD3.1 assembly that were represented via a SNP from the *Illumina® Bovine SNP50 v2* within 2500 bp. For both lists, the *H. sapiens* functional annotation was used due to the higher quality of the human genome annotation compared to bovine. P-values in PANTHER were calculated by a binomial statistical test and a Bonferroni correction was applied to account for multiple testing. Additionally, pathway analyses were carried out with the functional annotation tool in DAVID 6.7 [37] (http://david.abcc.ncifcrf.gov/home.jsp, accessed: 06/20/2013). The same reference list as for GO analysis using PANTHER was applied as background, and *H. sapiens* was again used for functional annotation.

In a last step, the roles of the *NCAPG* and *GDF8* genes within the growth network were further elucidated by building separate gene-gene interaction sub-networks for both genes. For this purpose, initially all genes were determined that had a significant partial correlation of additive gene effect with *NCAPG* effects in the PCIT growth network. *NCAPG*, these genes and their connections then formed a NCAPG-network, which was further analyzed with the Cytoscape plugin MCODE [38]. MCODE determines highly connected regions within networks using measurements of clustering coefficients and is suitable to detect gene-gene interaction complexes of high density. MCODE was run with default settings. For the analysis of *GDF8* and its connectivity in the growth network, an analogous procedure as for the NCAPG-network was applied.

## Results and discussion

### Genotyping & GWAS

In the present study, 152 male SEGFAM cattle were genotyped for 54,609 SNPs. Quality control with *Illumina® Genome Studio* excluded 2 animals due to call rates less than 0.98. Of the remaining 150 animals, weight measurements were available for 144 animals and metabolite measurements were available for 147 animals. After filtering for call rates and minor allele frequencies, the final SNP dataset for subsequent analyses comprised 44,505 high quality SNPs.

Single-trait-single-SNP GWAS were run for all 14 weight traits and 221 metabolites. Descriptive data and the results of the GWAS for the 13 phenotypes that were chosen for the AWM are presented in Table 1 and Additional file 1. The sample size in the present study was relatively small compared to other studies that performed GWAS on several hundreds or thousands of animals. Nevertheless, strong associations up to a significance level of 2.95 × 10^-7^ could be observed in the present GWAS, probably because the study took advantage of the specific design of the resource population. Namely, the application of embryo transfer techniques in establishing the population enabled the separation of systematic effects of maternal alleles on the intrauterine development from specific fetal allele effects on growth [6]. Furthermore, very uniform housing, feeding and sampling conditions within an experimental animal unit reduced the influence of environmental effects on the phenotypes. In addition, genotyping of sires and dams was helpful in detecting and reducing genotyping errors. Finally, the problem of population stratification, which might result in associations between phenotypes and unlinked candidate loci [39,40], could be controlled due to the populations’ F2 family pedigree. In sum, all these points controlled for systematic variability across the samples by standardizing for known effects and at least in part, might have compensated for the relatively small number of animals. The associations of anonymous SNPs from the 50k SNP chip are in agreement with recent association studies in the same population: trait-associated SNPs on BTA6 in the region of 30-40 Mb for tw273 and arginine (Additional file 1) are in accordance with previous studies where a polymorphism in *NCAPG* was found to be associated with growth and arginine metabolism in cattle [4-6]. For the metabolites PC_aa_C32:0, PC_ae_C36:1 and SM_C20:2, the SNP associations in the centromeric region of BTA2 (Additional file 1) might reflect the decreasing effects of *GDF8 Q204* on glycerophosphatidylcholines and sphingomyelins, which had been reported by Weikard and colleagues [6]. An overview of the most significant SNP for each of the 13 phenotypes that were chosen for the AWM is given in Table 2.

**Table 1:** Overview of phenotypic and genetic data

**Category**	**Trait**	**Acronym**	**N**	Mean*	Std. Dev.*	**p < 0.05**^**§**^	**p < 0.01**^**§**^	**p < 0.001**^**§**^
Weight	Total weight day 273	tw273	144	358.7	30.7	2744	697	90
Weight	Average daily gain day 183 - 273	dwg273	144	1.48	0.19	2504	674	98
Amino acid	Arginine	arg	146	93.0	23.0	2392	539	64
Amino acid	Lysine	lys	147	175.1	31.8	1935	400	48
Carnitine & Acylcarnitines	Free Carnitine	C0	147	6.1	0.84	1869	375	30
Carnitine & Acylcarnitines	Acetylcarnitine	C2	147	0.965	0.362	2049	393	38
Carnitine & Acylcarnitines	Valerylcarnitine	C5	147	0.064	0.018	2120	404	40
Carnitine & Acylcarnitines	Suberylcarnitine	C81	146	0.006	0.011	2360	514	74
Carnitine & Acylcarnitines	Myristylcarnitine	C14	147	0.011	0.004	2424	499	50
Carnitine & Acylcarnitines	Stearoylcarnitine	C18	147	0.021	0.011	2610	675	103
Glycerophosphatidylcholine	Diacylphosphatidylcholine C32:0	PC_aa_C32:0	146	4.72	1.65	5127	1221	79
Glycerophosphatidylcholine	Acylethylphoshatidylcholine C36:1	PC_ae_C36:1	146	13.4	5.07	2043	416	27
Sphingomyelin	Sphingomyelin C20:2	SM_C20:2	146	3.44	1.52	5487	1389	89

*Means and standard deviations for weight traits are given in [kg] and for metabolic traits in [μM].

§Number of SNPs in the GWAS with nominal p-values for association smaller than the indicated threshold as used for assembling the association weight matrix (AWM), total number of SNPs = 44505.

**Table 2:** The most significant SNP for each of the 13 growth network phenotypes

**Trait**	**SNP**	**Chromosome [UMD 3.1]**	**Position [bp]**	**p-value**
tw273	ARS-BFGL-NGS-66862	6	30585871	6.61 × 10^-6^
dwg273	ARS-BFGL-NGS-10175	8	57714648	3.39 × 10^-6^
Arginine	BTB-01456615	5	76659850	1.32 × 10^-5^
Lysine	BTB-01602960	5	88626757	2.45 × 10^-5^
C0	ARS-BFGL-NGS-41589	7	63843833	6.92 × 10^-5^
C2	ARS-BFGL-NGS-46431	28	36183465	2.82 × 10^-5^
C5	Hapmap51905-BTA-54176	22	35328236	1.26 × 10^-6^
C81	ARS-BFGL-NGS-117137	17	60340222	3.31 × 10^-7^
C14	BTA-121233-no-rs	1	94239208	5.75 × 10^-7^
C18	ARS-BFGL-BAC-4411	23	36070248	1.62 × 10^-6^
PC_aa_C32:0	Hapmap41889-BTA-49622	2	18108323	2.09 × 10^-5^
PC_ae_C36:1	Hapmap45612-BTA-28859	9	79191994	1.32 × 10^-5^
SM_C20:2	BTB-00081518	2	18408562	2.95 × 10^-7^

### AWM and PCIT

**Figure 2:**
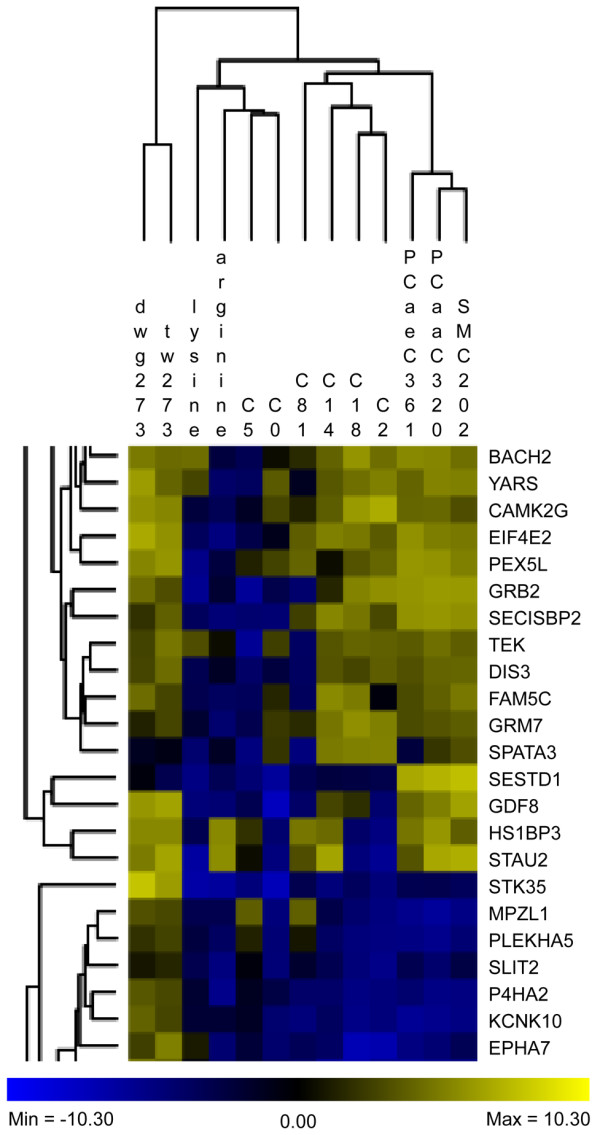
**Subset of the association weight matrix for growth at the onset of puberty.** Column wise, the AWM (association weight matrix) compares correlations between phenotypes, and row wise AWM compares gene-gene interactions. Cells within the matrix correspond to normalized additive effects of gene-associated SNPs as obtained from GWAS (genome-wide association studies). Squares of blue and yellow color gradients visualize the strength of standardized additive gene (SNP) effects. tw273: total body weight at month 9, dwg273: daily weight gain from month 6 to 9, C0: free carnitine, C2: acetylcarnitine, C5: valerylcarnitine, C81: suberylcarnitine, C14: myristylcarnitine, C18: stearoylcarnitine, PCaaC320: diacylphosphatidylcholine C32:0, PCaeC361: acylethylphoshatidylcholine C36:1, SMC202: sphingomyelin C20:2.

**Figure 3:**
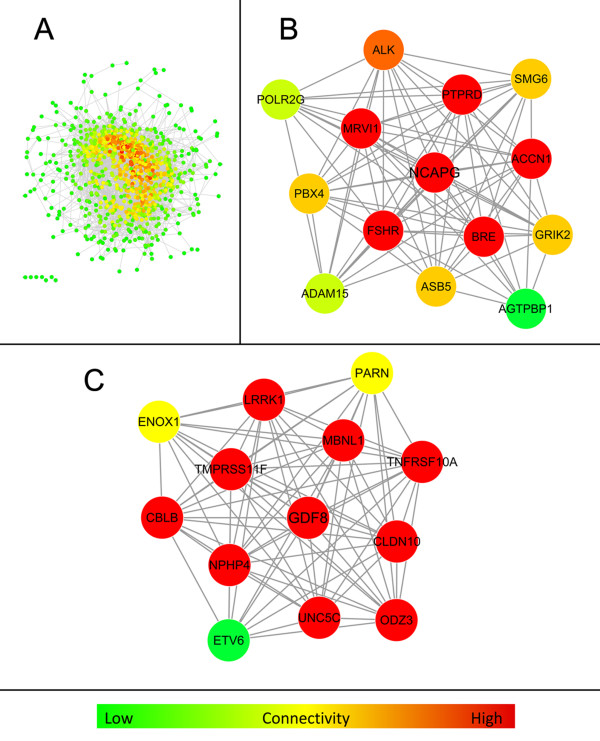
**Gene networks for growth at the onset of puberty.** Each node in the networks represents a gene with at least one significant partial correlation of additive effects to another gene in the network as identified by Partial correlation information theory (PCIT) from the Association weight matrix (AWM). Edges represent significant interactions between genes. Node colors provide gradual information about the number of connections of a specific node in the respective network. The color scale ranges from green (few connections) over yellow (some connections) to red (many connections). **(A)** Growth at onset of puberty network. **(B)** NCAPG sub-network established from the full growth network after identifying the densest subcluster using MCODE software. **(C)** GDF8 sub-network established analogous to the NCAPG sub-network.

Given the direct or indirect relevance of the key and supportive traits for mammalian growth, the AWM approach identified genes critical for growth at the onset of puberty from GWAS results. A main advantage of this approach over single-trait-single-SNP analyzes is the simultaneous information captured from a collection of phenotypes. This approach ends up with a set of genes and interactions potentially affecting the complex trait that would be overlooked in single-SNP-single-trait analyzes (as demonstrated in [23]). Based on standardized additive SNP effects, the AWM explores column wise and row wise the relationships among phenotypes and additive gene effects, respectively. In accordance with the four different classes of traits (weights, amino acids, acylcarnitines, phospho- & sphingolipids), the traits split in four distinct clusters in our AWM (Figure 2). The first cluster comprises the two weight traits, the second the amino acids, carnitine and a short chain acylcarnitine, the third the medium to long chain acylcarnitines and acetylcarnitine, and the fourth cluster contains the phospho- & sphingolipids. This result from standardized SNP-associated effects is in line with data from the heat map analysis of raw phenotypic correlations (Figure 1), where the amino acids and phospho- & sphingolipids formed two distinct clusters, whereas the acylcarnitines have been found to be a more heterogeneous group. The AWM served as input for the PCIT algorithm which identified significant gene-gene interactions with impact on weight traits and metabolomic traits. PCIT therefore determined nodes and edges for the growth network (Figure 3A) where each node is a putatively relevant gene for growth at onset of puberty and each edge displays a significant interaction between two genes. Based on the partial correlations of standardized additive gene effects, PCIT determined 964 genes out of 985 AWM-genes to be significantly partially correlated with at least one other gene. In total, PCIT detected 11,894 undirected interactions between these 964 genes.

### Growth network

The present study examined the genetic and metabolic background for divergent growth in the SEGFAM resource population. The interval comprising the onset of puberty was chosen as an observation period, because this is the key interval for differential growth in cattle [41]. A data set comprising metabolic, physiological and genetic data was analyzed using a systems biology approach. With the help of this approach, a gene interaction network was constructed and subsequently analyzed with data mining tools in order to reveal genes and pathways that are presumably involved in physiological processes that lead to differential growth. The derived growth network comprised of 964 genes (or nodes) connected by 11,894 edges (Figure 3A). To test if the numbers of genes and interactions in this network were higher than expected by chance, random gene-gene interaction networks were built and compared with the growth network. On average, the random networks comprised 771 genes that were connected by only 1,010 edges (Additional file 2). This massive decrease in the number of edges in the random networks is in agreement with recent literature [23].

**Figure 4:**
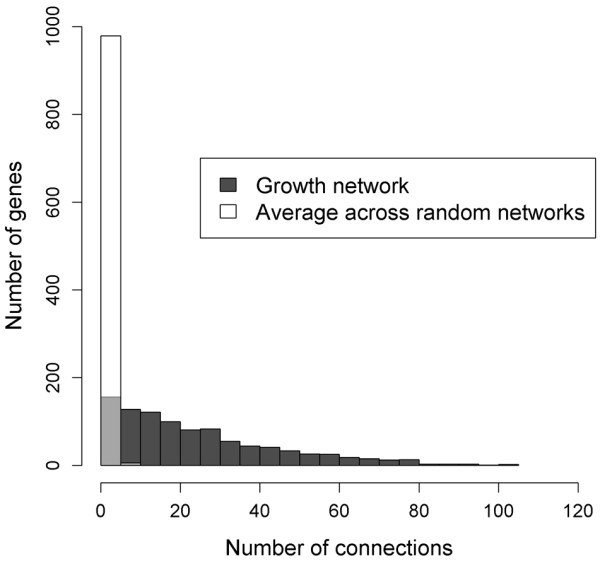
**Comparison of connections per gene in the growth-network versus the average number of connections per gene across all random networks.** The figure illustrates the number of connections per gene in the growth network and the average number of connections per gene across the random networks. Due to the transparent style of the white bars, black bars or parts of black bars that are hidden by a white bar are colored in light grey. The respective detailed data are provided in Additional file 3.

Random or “non-biological” networks are characterized by nodes, which all possess nearly the same small number of edges [42]. In contrast to this, “real” or biological networks typically display a scale-free structure, which is characterized by many lowly and only a few highly connected nodes [43]. This criterion is fulfilled by the growth network, as the majority of nodes is only weakly connected with other nodes, whereas a few nodes are highly connected (Figure 4, for highly connected nodes see Table 3). Comparing the number of connections per node between the growth network and the random networks underpins the growth network’s non-random nature: nodes in the growth network are connected to up to 105 other nodes, whereas nodes in the random networks are connected to a maximum of only 18 other nodes (Figure 4, Additional file 3). Thus, the topology of the growth network and the random networks substantially differ due to the following reasons: (i) Genes in the growth network display a much higher variability concerning their number of connections to other genes than genes in the random networks, and (ii) genes in the growth network are connected up to more than 100 other genes, whereas genes in the random networks usually show no more than 5 connections to other genes on average.

**Table 3:** The 10 most densely connected genes within the gene-gene interaction network

ID*	Official gene name*	**Chromosome**	**Position [UMD 3.1]**	**Connectivity**^**§**^
*TH1L*	TH1-like (Drosophila)	13	57899212 - 57909996	105
*BTC*	Betacellulin	6	91430305 - 91480129	104
*MRVI1*	Murine retrovirus integration site 1 homolog	15	42548308 - 42674631	100
*USP40*	Ubiquitin specific peptidase 40	3	113786837 - 113866952	93
*FHIT*	Fragile histidine triad	22	41319551 - 42108622	92
*DGKH*	Diacylglycerol kinase eta	12	12293695 - 12405237	91
*DNAJB14*	DnaJ (Hsp40) homolog, subfamily B, member 14	6	26105476 - 26143561	87
*BRE*	Brain and reproductive organ-expressed (TNFRSF1A modulator)	11	71403125 - 71826561	87
*STX12*	Syntaxin 12	2	126134951 - 126170517	86
*PRLHR*	Prolactin releasing hormone receptor	26	39219423 - 39220535	83

*In accordance with HUGO Gene Nomenclature Committee (HGNC) definitions.

§Total number of genes to which the target gene is connected in the gene-gene interaction network via a direct edge.

Taken together, these results suggest that the growth network is a non-random network and that our approach was able to predict gene-gene interactions with a frequency higher than could be achieved by chance alone.

In order to explore how much of the information in the growth network was due to including metabolomic data in the analysis, a network containing solely metabolomic data was created. The resulting network comprised 116 genes (or nodes) which were connected by 1767 edges. Further analyses revealed that 104 of these genes and 301 of these edges were also present in the growth network. These numbers are substantially smaller than the total number of genes and edges in the full growth network. Thus, we can conclude that only a minor part of the growth network is due to metabolomic traits only and has no correlations to the key traits. The remaining genes and gene-gene interactions in the network should be due to data from physiological traits only or from the combined information from physiological traits and metabolomic traits. An equivalent analysis restricted to the physiological growth data, however, was not possible, because the correlation-based inference as performed by PCIT cannot be implemented with only 2 traits (all correlations would be either +1 or −1). As a consequence, the proportion of contribution from those two sources to the full growth network cannot be quantified.

**Figure 5:**
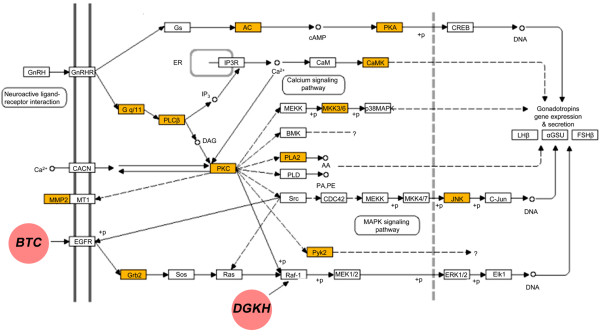
**Gonadotropin-releasing hormone (GnRH) signaling pathway containing genes that are represented in the Partial Correlation Information Theory (PCIT) network “growth at the onset of puberty”**. DAVID analysis indicating a nominally significant enrichment of genes from the GnRH signaling pathway that are associated with key- or supportive phenotypes for growth at the onset of puberty. Pathway components that are encoded by genes included in the PCIT network are colored in orange. Purple dots highlight betacellulin (BTC) and diacylglycerol kinase eta (DGKH), which were identified as major hubs in the growth network (Table 3). Arrows indicate molecular interactions or relations, dotted arrows indicate indirect effects. Graph adapted from the Kyoto Encyclopedia of Genes and Genomes (KEGG) (http://www.genome.jp/kegg-bin/show_pathway?hsa04912, accessed: 06/20/2013).

Subsequently, the growth network was tested for its biological relevance via tests for overrepresentation of specific biological processes and molecular pathways. Sorted by decreasing specificity, gene ontology (GO) analyses revealed significant overrepresentations of genes acting in “cell surface receptor signaling pathways”, “signal transduction”, “cell communication”, “cell adhesion” and “cellular processes” (Table 4). These results reflect the current literature, because complex signaling events between cells and tissues are known to be crucial for growth in mammals [44]. Pathway analyses with DAVID revealed enrichments in the KEGG pathways “GnRH signaling” (p-value = 5.0 × 10^-3^), “vascular smooth muscle contraction” (p = 2.8 × 10^-2^) and “gap junction” (p = 2.3 × 10^-2^), although the p-values were no longer significant when corrected for multiple testing. From this list, the components of the GnRH signaling pathway were of particular interest, because GnRH signaling triggers sexual maturation in a number of species including male cattle [45-47]. Pulsatile releases of GnRH from the hypothalamus cause the release of luteinizing hormone (LH) and follicle stimulating hormone (FSH) from the anterior pituitary gland, which in turn is necessary for spermatogenesis and maturation. GnRH signaling exerts substantial effects on growth at the onset of puberty in mammals as demonstrated by Yingling *et al*. [48,49]. In their studies, the authors delayed the onset of puberty in rats by GnRH-antagonist injections into the hypothalamus. This treatment had major impact on body weight and bone development in the treated animals. We therefore conclude that the divergent growth at puberty in the SEGFAM population is extensively mediated by components from the GnRH signaling cascade, because a high number of genes encoding components in the GnRH signaling pathway are represented in the growth network (Additional file 4, Figure 5). Important processes like the activation of mitogen-activated protein kinases, calcium release via PLCβ (phospholipase C, beta) or cAMP regulation via Gs (guanine nucleotide-binding protein G) and AC (adenylate cyclase) are affected by these genes. Interestingly, *BTC* and *DGKH*, two highly connected nodes (hubs) in the growth network, encode proteins that are established binding partners of components in the GnRH signaling pathway (Table 3, Figure 5). Besides their structural importance (hubs link the less connected nodes to the whole network), hubs in scale-free networks also tend to be good predictors for the biological processes within the network [50,51]. *BTC* belongs to the epidermal growth factor (EGF) family which stimulates growth, proliferation, and differentiation of cells [52]. BTC has been reported to bind to the EGF receptor and to have a mitogenic and growth promoting effect on mesenchymal cells [53]. In the present study, effects of BTC on divergent growth might be mediated by its interactions with the epidermal growth factor receptor (EGFR) (Figure 5). Watanabe *et al*. confirmed EGFR as the primary receptor for BTC [54]. Studies on ovarian follicles detected an interplay between BTC and LH in follicle maturation [55,56]. It was concluded that BTC is a downstream mediator of LH and propagates LH signals. LH is essential for the synthesis of testosterone production, which has a variety of effects during sexual maturation and development. Due to the physiological interplay between BTC and LH, and *BTCs’* outstanding position as a hub in the growth network, we put up the hypothesis that *BTC* might act as a trigger of growth in our resource population. We assume that BTC might exert its effects on divergent growth by interacting with genes from the initial steps of the GnRH cascade as proposed in Figure 5. Downstream interactions with LH might finally affect testosterone metabolism, which is a known driver of growth at puberty *in vivo*.

**Table 4:** **Significantly enriched biological processes within the gene-gene interaction network, estimated by GO term enrichment analyses using *PANTHER 8.0***

**Biological process**	N reference*	**N network (observed)**^**§**^	**N network (expected)**^**#**^	***P *** **value**^**+**^
Cell communication	1982	347	271	8.60E-06
Signal transduction	1865	325	255	5.25E-05
Cellular process	2742	450	375	6.33E-05
Cell surface receptor signaling pathway	893	169	122	1.45E-03
Cell adhesion	638	126	87	3.97E-03

*Total number of genes in the reference list for the respective biological process.

§Total number of genes in the gene-gene interaction network for the respective biological process.

#Total number of number of genes that would be expected by chance.

+Significance of enrichment calculated by a binomial statistical test and corrected for multiple testing via a Bonferroni correction.

The results of the present study suggest that diacylglycerol kinase eta (*DGKH*) might be a second candidate gene for interactions affecting divergent growth in the resource population. Analogous to *BTC*, *DGKH* is also one of the major hubs in the growth network and is therefore likely implicated in interactions modulating growth (Table 3). DGKH is a member of the enzyme family of diacylglycerol kinases, which catalyze the phosphorylation of diacylglycerol to produce phosphatidic acid (reviewed in [57]). DGKH was proposed as a regulator protein of the Ras/Raf/MEK/ERK signaling cascade, because it targets and activates Raf (especially C-Raf) [58]. This cascade controls a vast number of growth regulating processes in cells, including proliferation, transformation and differentiation [59]. The overall importance of C-Raf for proper development and growth is impressively demonstrated by a study on C-Raf mutant mice [60]. In this study, Raf mutant individuals either died because of severe developmental defects or showed distinct growth retardations. Therefore, DGKH might interact with growth processes via its activating properties on C-Raf, which might subsequently modify growth via its downstream signaling partners. Based on our results and the supporting literature, we therefore propose *DGKH* as a modulator of growth in our resource population. In contrast to *BTC*, which might trigger the initial steps of the GnRH cascade, *DGKH* exerts its effects further downstream in GnRH signaling (Figure 5). We therefore propose an interaction model in which divergent growth is induced by *BTC* signals which might be modulated by *DGKH*.

### NCAPG-network

The *NCAPG/LCORL* locus seems to be an important conserved modulator of mammalian pre- and postnatal growth, because it previously had been identified in GWAS within several populations and species including human [12,17,61,62]. *NCAPG* is a potential effector of a QTL on BTA6 affecting pre- and postnatal growth in our resource population, with the most pronounced *NCAPG* effects on body growth being observed at the onset of puberty [4-6]. Thus, the role of *NCAPG* and its interactions with other genes from the growth network was of specific interest and examined in a NCAPG-specific sub-network. NCAPG belongs to the family of condensins and is an important mediator for chromosome condensation during mitosis, where it interacts with DNA methyltransferase DNMT3B [63,64]. However, the physiological pathways through which *NCAPG* affects body weight at onset of puberty are largely unknown. Recently, Weikard *et al*. [6] described associations between *NCAPG* variants and serum levels of arginine, which suggests a role of *NCAPG* in arginine metabolism. As arginine metabolism is involved in lipid metabolism, growth and developmental processes in mammals [65], this result represents a promising indicator of the physiological background of *NCAPG*. However, a conclusive physiological link between the role of NCAPG during mitosis, the effect on arginine level and pre- and postnatal growth is still missing. To further elucidate this relationship, the present study established a gene-gene interaction network comprising *NCAPG* and its densely interacting genes from the growth network. The aim was to detect genes with strong partially correlating additive effects on growth, which thus might complement *NCAPG* functions in divergent growth. An NCAPG specific network restricted to *NCAPG* and all genes that were connected to *NCAPG* in the growth network contained 37 different genes (Table 5). In contrast to the high connectivity of *NCAPG* in this growth-derived network, *NCAPG* was connected to an average of only 2.3 genes in the random-networks, thus underpinning the non-random nature of *NCAPG* connections in the growth network (Additional file 3). In the second step, the NCAPG–specific network was extracted for genes that most densely interact with each other by using the MCODE software. The resulting final NCAPG sub-network comprises the following 14 genes (Figure 3B, Table 5, connections provided in parentheses): *ACCN1* (13 connections), *BRE* (13), *FSHR* (13), *MRVI1* (13), *NCAPG* (13), *PTPRD* (13), *ALK* (12), *ASB5* (11), *GRIK2* (11), *PBX4* (11), *SMG6* (11), *ADAM15* (10), *POLR2G* (10), and *AGTPBP1* (8).

**Table 5:** The genes in the NCAPG-specific networks

				**Connectivity**	
**GeneID**	Official gene name*	**BTA**^**†**^	**Position [UMD 3.1]**	**NCAPG-specific sub-network (MCODE derived)**^**§**^	**NCAPG-specific network**^**§**^
ACCN1	Acid-sensing (proton-gated) ion channel 2	19	16353062 - 17563070	13	21
ADAM15	ADAM metallopeptidase domain 15	3	15593181 - 15603336	10	23
AGTPBP1	ATP/GTP binding protein 1	8	80235082 - 80433151	8	13
ALK	Anaplastic lymphoma receptor tyrosine kinase	11	70321848 - 70646314	12	15
ARNTL2	Aryl hydrocarbon receptor nuclear translocator-like 2	5	82875251 - 82956797	0	9
ASB5	Ankyrin repeat and SOCS box containing 5	27	6692312 - 6736965	11	15
BRE	Brain and reproductive organ-expressed (TNFRSF1A modulator)	11	71403124 - 71826561	13	18
CAPN2	Calpain 2, (m/II) large subunit	16	27781671 - 27840011	0	11
DNAJC2	DnaJ (Hsp40) homolog, subfamily C, member 2	4	44769574 - 44803651	0	4
Drosophila	Single-minded homolog 1 (Drosophila)	9	50160697 - 50228919	0	12
ELMO1	Engulfment and cell motility 1	4	60356210 - 60838995	0	14
ELOVL5	ELOVL fatty acid elongase 5	23	25155742 - 25228997	0	37
FSHR	Follicle stimulating hormone receptor	11	31110744 - 31305197	13	16
GRIK2	Glutamate receptor, ionotropic, kainate 2	9	48857302 - 48925683	11	14
IGSF21	Immunoglobin superfamily, member 21	2	134864753 - 135060996	0	8
INSR	Insulin receptor	7	17279726 - 17421470	0	7
ITPKB	Inositol-trisphosphate 3-kinase B	16	30390092 - 30491749	0	12
JAZF1	AZF zinc finger 1	4	68444035 - 68773107	0	14
LPL	Lipoprotein lipase	8	67497758 - 67511231	0	4
MRVI1	Murine retrovirus integration site 1 homolog	15	42548308 - 42674631	13	22
MXD4	MAX dimerization protein 4	6	108490181 - 108501583	0	13
NCAPG	Non-SMC condensin I complex, subunit G	6	38765969 - 38812056	13	37
p600	Interleukin 13	7	23018546 - 23020546	0	11
PBX4	Pre-B-cell leukemia homeobox 4	7	3631055 - 3685738	11	17
PDE4D	Phosphodiesterase 4D, cAMP-specific	20	18748587 - 20322583	0	10
PLCB4	Phospholipase C, beta 4	13	2211089 - 2411116	0	15
POLR2G	Polymerase (RNA) II (DNA directed) polypeptide G	29	41777025 - 41780284	10	17
PPP1R3A	Protein phosphatase 1, regulatory subunit 3A	4	54866421 - 54906488	0	6
PTPRD	Protein tyrosine phosphatase, receptor type, D	8	36342559 - 36800975	13	27
RAB6IP1	RAB6 interacting protein 1	15	43965508 - 44059813	0	12
RBMS3	RNA binding motif, single stranded interacting protein 3	22	3898961 - 4695095	0	7
RGS7	Regulator of G-protein signaling 7	16	36478831 - 36695721	0	12
SCPEP1	Serine carboxypeptidase 1	19	8018816 - 8052591	0	8
SMG6	Smg-6 homolog, nonsense mediated mRNA decay factor (C. elegans)	19	23660756 - 23858481	11	16
SPAG9	Sperm associated antigen 9	19	36343607 - 36422983	0	1
THSD7A	Thrombospondin, type I, domain containing 7A	4	19451228 - 19727594	0	14
ZBTB16	Zinc finger and BTB domain containing 16	15	24917133 - 25116350	0	10

†Bos taurus chromosome.

*In accordance with HUGO Gene Nomenclature Committee (HGNC) definitions.

§Total number of genes to which the target gene is connected via a direct edge.

**Figure 6:**
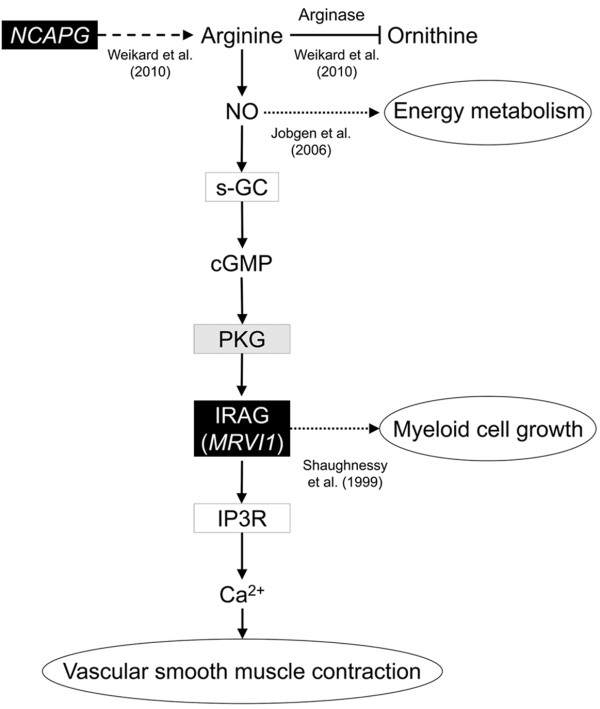
**Model for mechanisms of divergent growth associated with NCAPG.** Black boxes identify genes and gene products that interact with each other in the NCAPG-network (Figure 3B). Grey shaded boxes specify gene products which are encoded by genes from the global growth network (Figure 3A). Arrows indicate molecular interactions, dashed arrows indicate genetic effects, dotted lines indicate physiological effects and blocked lines indicate a decreased pathway activity. Graph adapted from [6] and the Kyoto Encyclopedia of Genes and Genomes (KEGG) (http://www.genome.jp/kegg/, pathway: vascular smooth muscle contraction, accessed: 06/20/2013).

Interestingly, murine retrovirus integration site 1 homolog (*MRVI1*, also known as *IRAG*), one of the major hubs in the growth network (Table 3), is also among the most highly connected genes in the NCAPG sub-network (Table 5). Thus, we suggest that *MRVI1* might play an important role in divergent growth due to its possible interactions with *NCAPG* in a common pathway. *MRVI1* controls physiological functions that depend on nitric oxide (NO) [66,67], which in turn needs arginine as a substrate (Figure 6). NO is a signaling molecule that exerts its effects in a wide range of metabolic processes, and it is generally crucial for the maintenance of energy homeostasis in mammals (as reviewed in [68]). Its effects on growth might be mediated by its properties in glucose and lipid metabolism as well as the turnover of proteins. Because the effects of *MRVI1* effects on body growth strongly correlate with *NCAPG* effects (as proven with the NCAPG sub-network), we argue that both genes might influence body growth by the arginine-NO pathway. This suggests a physiological role of *NCAPG* in NO signaling, additionally to its supposed role in arginine metabolism. Interestingly, Weikard *et al*. [6] concluded from their data that the observed different serum arginine levels in the individuals from the resource population might result from a decreased arginase activity (arginase converts arginine to ornithine). Because arginase and NOS (NOS converts arginine to NO) compete for cellular arginine as substrate, an inhibition of arginase activity might favor NO synthesis (Figure 6). Therefore, our results support the hypothesis of Weikard *et al*. and indicate that *NCAPG* might indirectly affect the NO pathway through its effects on arginine metabolism. NO and *MRVI1* are implicated in the contraction of the vascular smooth muscle, which regulates the blood flow and subsequently the nutrient supply in peripheral tissues. This process is achieved by a signaling cascade, through which NO and MRVI1 reduce intracellular Ca^2+^ concentrations. Thus, *NCAPG* and *MRVI1* might interactively modulate growth through their effects on arginine-NO dependent vascular smooth muscle contractions (Figure 6). Schlossmann *et al*. [67] assume that the established regulatory effects of *MRVI1* on intracellular Ca^2+^ supply also could directly contribute to cell growth, because Ca^2+^ impacts cell vitality. Taken together, our results indicate interactions of *NCAPG* and *MRVI1* in cattle growth, which presumably might be mediated by the complementary effects *of NCAPG* and *MRVI1* in the arginine-NO metabolism. Regarding potential NO effects via vascular smooth muscle contraction, it is interesting that BTC, another major hub in our growth network, is a potent mitogen of vascular smooth muscle cells [69]. Vascular smooth muscle cells interact with endothelial cells to enable formation of new blood vessels [70] which is essential for an appropriate blood supply in growing tissue.

Besides the *NCAPG* – *MRVI1* correlation of additive effects, we further detected connections between *NCAPG*, follicle stimulating hormone receptor (*FSHR*) and glutamate receptor, ionotropic, kainate 2 (*GRIK2*) in the NCAPG sub-network. *FSHR* and *GRIK2* play important physiological roles in maturation and puberty. FSHR is the receptor for follicle stimulating hormone (FSH), which is important for the development of reproductive organs during puberty [71]. GRIK2 belongs to the kainate family of glutamate receptors, which transmit glutamate induced signaling events. Glutamate is one of the most important signaling molecules in the brain, and it is of specific relevance for the onset of puberty, because glutamergic neurotransmission initiates GnRH release. Interactions of *NCAPG*, *FSHR* and *GRIK2* in the NCAPG-network, due to partial correlations of additive gene effects on growth at the onset of puberty, thus suggest a possible indirect connection between *NCAPG*, growth at puberty and regulation of reproductive functions in the investigated bovine resource population.

### GDF8-network

GDF8 is a major regulator of pre- and postnatal growth and specifically muscle development in many vertebrate species including mice, dog, sheep, cattle, horse and human [7,10,11,16,18]. Several mutations in *GDF8* have been shown to cause the muscular hypertrophy phenotypes in cattle [7,9]. *GDF8* negatively regulates muscle development by limiting the growth of muscle fibers. Thus, *GDF8* is directly involved in processes that contribute to mammalian body growth. The SEGFAM resource population segregates for the *GDF8 Q204X* mutation, and pronounced effects of *GDF8 Q204X* on body mass gain, protein accretion and free plasma carnitine had been observed in this population at the onset of puberty [6]. Consequently, the growth network was examined for genes that cluster densely together with *GDF8* in order to generate a network that captures information about gene-gene interactions of *GDF8* in cattle growth at the onset of puberty. Analogously to the NCAPG-network, we first extracted a GDF8-specific network from the total growth network. Subsequently, the resulting set of genes and their interactions were examined with MCODE in order to identify the highly connected regions within this network. In the resulting GDF8 sub-network, *GDF8* was connected to 12 other genes (Table 6). In contrast, *GDF8* was connected to only 3.7 genes on average in the random networks. This observation underlines the non-random nature of the GDF8 sub-network.

**Table 6:** The genes in the GDF8-specific sub-network

**GeneID**	Official gene name*	**Chromosome**	**Position [UMD 3.1]**	**Connectivity**^**§**^
CBLB	Cas-Br-M (murine) ecotropic retroviral transforming sequence b	1	50659277 - 50880121	12
CLDN10	Claudin 10	12	76758754 - 76864806	12
ENOX1	Ecto-NOX disulfide-thiol exchanger 1	12	13381372 - 13679287	11
ETV6	Ets variant 6	5	98412609 - 98576590	10
GDF8	Myostatin	2	6213566 - 6220196	12
LRRK1	Leucine-rich repeat kinase 1	21	5642545 - 5785655	12
MBNL1	Muscleblind-like splicing regulator 1	1	116238830 - 116394390	12
NPHP4	Nephronophthisis 4	16	48181539 - 48314477	12
ODZ3	Odz, odd Oz/ten-m homolog 3 (Drosophila)	27	12356887 - 12807702	12
PARN	Poly(A)-specific ribonuclease	25	13455797 - 13634775	11
TMPRSS11F	Transmembrane protease, serine 11 F	6	85473854 - 85612662	12
TNFRSF10A	Tumor necrosis factor receptor superfamily, member 10a	8	71177822 - 71189201	12
UNC5C	Unc-5 homolog C (C. elegans)	6	30361298 - 30787250	12

*In accordance with HUGO Gene Nomenclature Committee (HGNC) definitions.

§Total number of genes to which the target gene is connected in the NCAPG-subcluster via a direct edge.

Muscle development and remodeling are complex events that depend on a wide range of transcription factors, signaling molecules, metabolites and proteins (reviewed in [72]). This is in agreement with the GDF8 sub-network containing a diverse set of genes involved in a variety of pathways and processes linked to muscle development. Interestingly, leucine-rich repeat kinase 1 (*LRRK1*) is one of the genes present in the GDF8 sub-network. LRRK1 regulates trafficking of EGFR in the endosomal system and is therefore involved in the recycling and degradation of EGFR [73]. EGFR processes EGF induced signaling and stimulates growth processes in a variety of tissues as outlined above for the entire growth network (Figure 5). Thus, *LRRK1* links the global growth network with the specific GDF8 sub-network and might contribute to body and muscle growth in the SEGFAM resource population via its impact on EGFR turnover. In summary, the GDF8 sub-network underpins the complex interplay of transcription and splicing factors, signaling molecules and decay regulators in growth and muscle remodeling.

## Conclusions

To our knowledge our study is the first to combine genetic, metabolomic and physiological data in a systems biology approach in cattle. This innovative approach was able to obtain valuable new insight into the genetic background of divergent growth in cattle. Our data indicate GnRH signaling as a relevant genetic modulator of bovine growth at the onset of puberty. In addition to the confirmed effects of two conserved mammalian growth modulators, *NCAPG* and *GDF8,* our data suggest that *BTC* and *DGKH* might be further mediators of divergent growth in our cattle resource population via gene-gene interactions. Our data support the existing assumptions about the physiological role of *NCAPG* in arginine-NO metabolism and propose a model, in which downstream processes of NO signaling, including *MRVI1* effects, might act on divergent growth. Furthermore, we obtained indication of an indirect connection between growth at puberty and regulation of reproductive functions due to *FSHR* and *NCAPG* interactions. Further studies will investigate the regulation of the highlighted genes and pathways to obtain advanced data on their specific role in divergent growth at the onset of puberty.

## Competing interests

The authors declare that they have no competing interests.

## Authors’ contributions

PW contributed to data collection, carried out the data analysis, interpreted the data and prepared the manuscript. AR performed the data analysis, participated in data interpretation and finally revised the draft manuscript. MF contributed to data analysis and interpretation. RW contributed to data interpretation and finally revised the draft manuscript. KS, HH and EA contributed to data collection. CK conceived the study, contributed to data collection, participated in data interpretation and finally revised the draft manuscript. All authors read and approved the final manuscript.

## Supplementary Material

Additional file 1 **Manhattan plots of GWAS results.** Significance of association (−log_10_(p)) between SNPs across the bovine genome and the target traits from the groups of body weights (A,B), amino acids (C,D), acylcarnitines (E-J), phosphatidylcholines (K,L) and sphingomyelins (M) (N = 144 – 147). (A) total weight at month 9 (tw273), (B) daily weight gain from month 6 to 9 (dwg273), (C) arginine, (D) lysine, (E) free carnitine (C0), (F) acetylcarnitine (C2), (G) valerylcarnitine (C5), (H) suberylcarnitine (C8:1), (I) myristylcarnitine (C14), (J) stearoylcarnitine (C18) (K), diacylphosphatidylcholine C32:0 (PC_aa_C32:0), (L) acylethylphoshatidylcholine C36:1 (PC_ae_C36:1), (M) sphingomyelin C20:2 (SM_C20:2). Click here for file

Additional file 2 **Number of connections per gene for the growth network, the metabolites-only network and for each of the 10 random networks.** The table summarizes the number of connections per gene as determined from the gene-gene interaction network as determined by PCIT for the three different kinds of networks (growth network, metabolites-only network and random networks). Click here for file

Additional file 3 **List of the genes in the association weight matrix (AWM) and their number of connections in the growth network, the metabolites-only network and in each of the 10 random networks.** The table lists all genes included in the AWM and provides the total number of connections for each of the genes in the growth network, the metabolites-only network and in each of the 10 random networks. Click here for file

Additional file 4 Components of the Gonadotropin releasing-hormone pathway that are encoded by genes from the growth network. Click here for file
