# Supplementary material for: A systems biology approach using metabolomic data reveals genes and pathways interacting to modulate divergent growth in cattle
Source: BMC Genomics. 2013 Nov 18;14:798. doi: 10.1186/1471-2164-14-798 (PMC3840609; doi:10.1186/1471-2164-14-798)

Additional_file_1: Manhattan plots of GWAS results. Shown is the significance of association (-$\log_{10}(p)$) between SNPs across the bovine genome and the target traits from the groups of body weights (A,B), amino acids (C,D), acylcarnitines (E-J), phosphatidylcholines (K,L) and sphingomyelins (M) (N = 144 – 147). The red line marks the significance level of p = 0.05 before correction for multiple testing. SNPs above this line theoretically had the chance to be represented via a gene in the AWM.

(A) total weight day 273 (tw273), (B) average daily gain day 183–273 (dwg273), (C) arginine, (D) lysine, (E) free carnitine (C0), (F) acetylcarnitine (C2), (G) valerylcarnitine (C5), (H) suberylcarnitine (C8:1), (I) myristylcarnitine (C14), (J) stearoylcarnitine (C18) (K), diacylphosphatidylcholine C32:0 (PC_aa_C32:0), (L) acylethylphoshatidylcholine C36:1 (PC_ae_C36:1), (M) sphingomyelin C20:2 (SM_C20:2).

(A) tw273

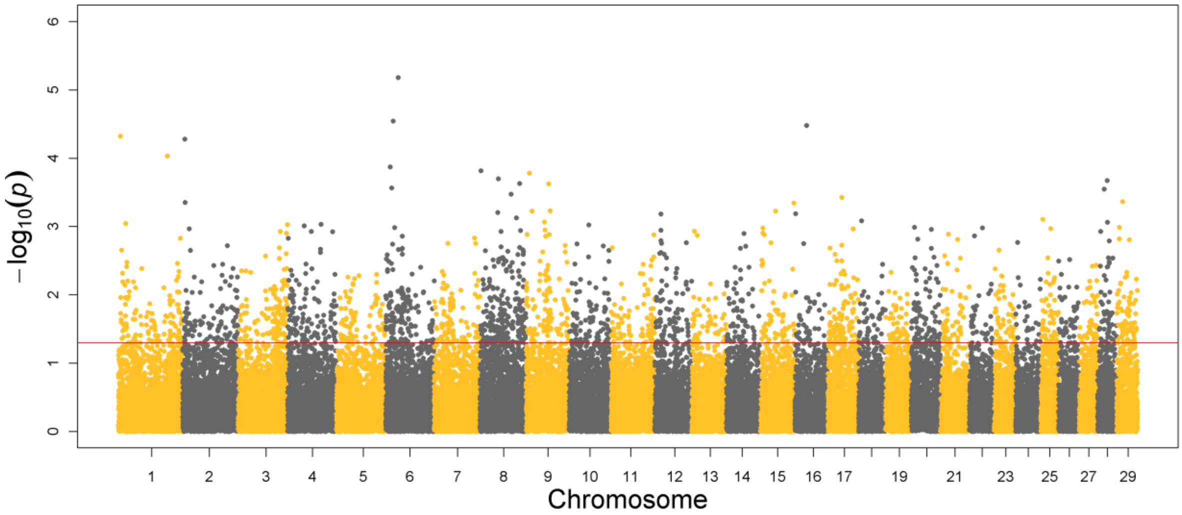

(B) dwg273

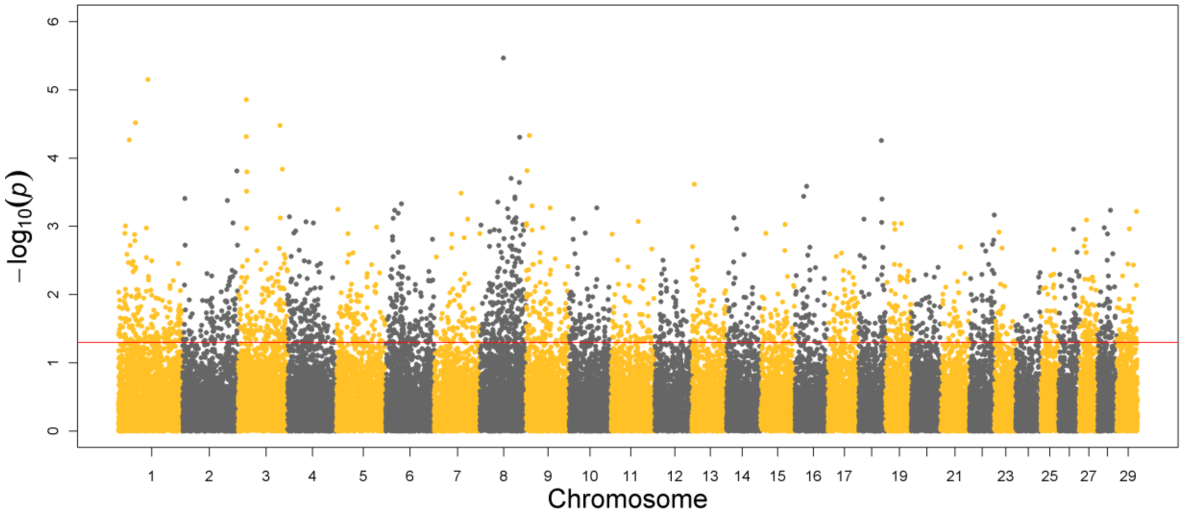

(C) Arginine

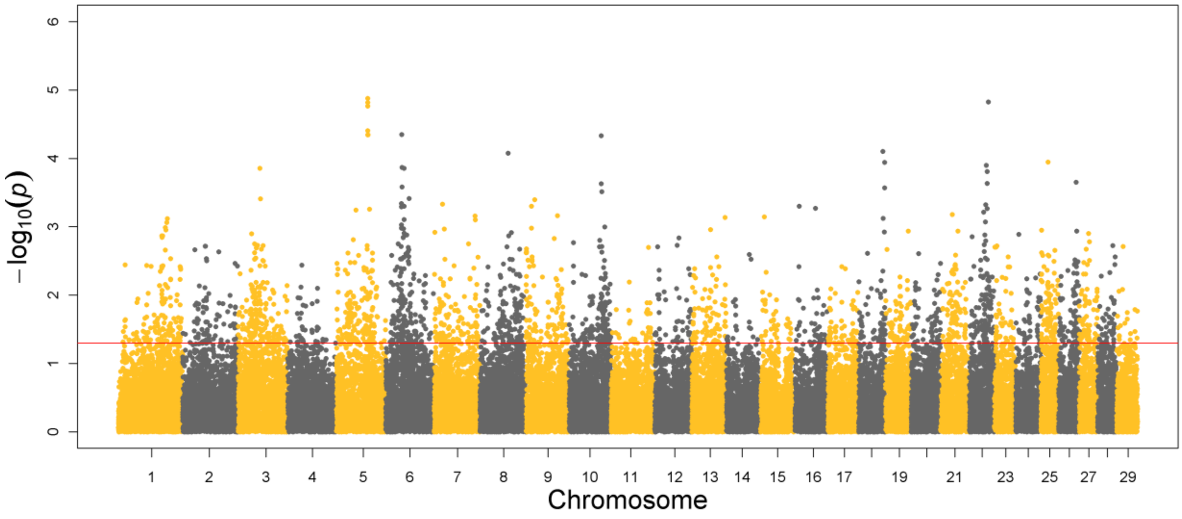

(D) Lysine

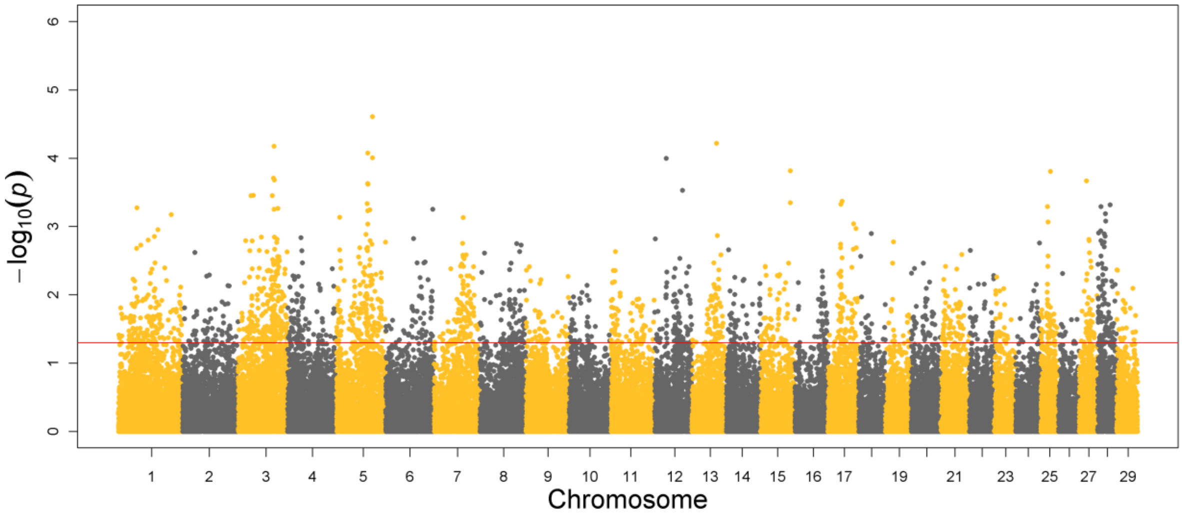

(E) C0

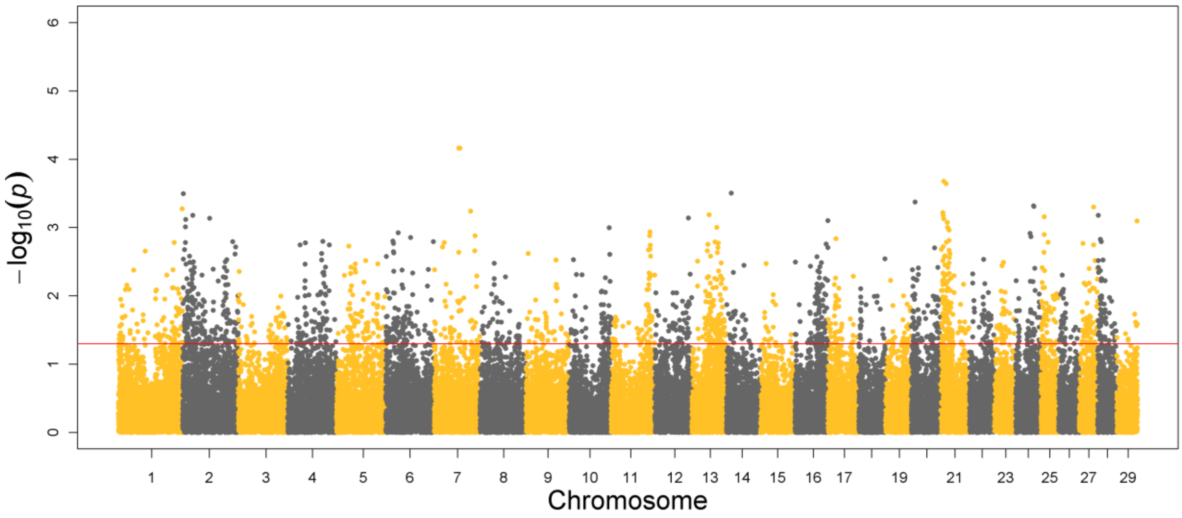

(F) C2

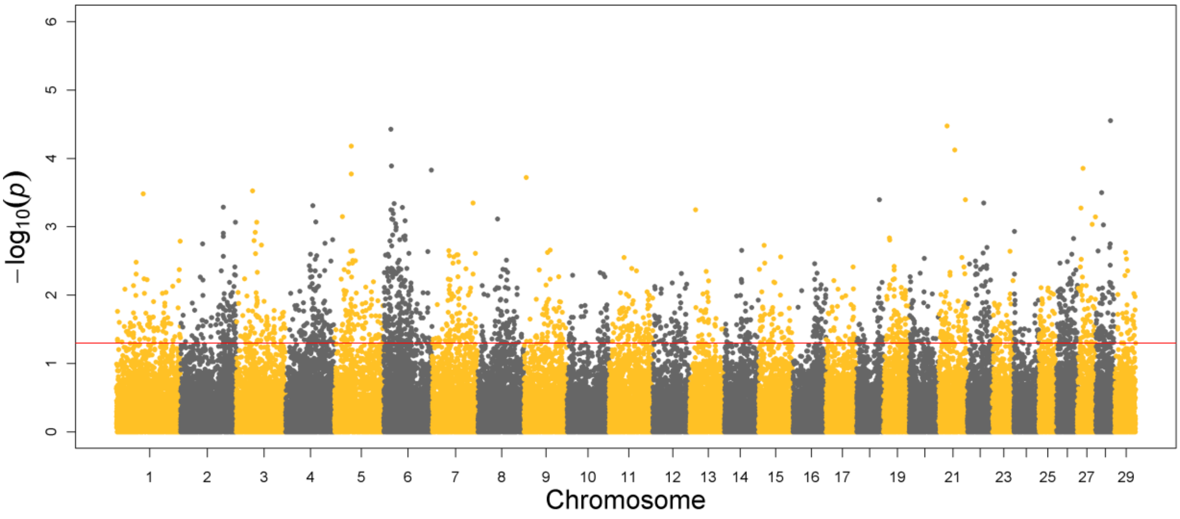

$-\log_{10}(p)$
0 1 2 3 4 5 6
1 2 3 4 5 6 7 8 9 10 11 12 13 14 15 16 17 19 21 23 25 27 29
Chromosome

(G) C5

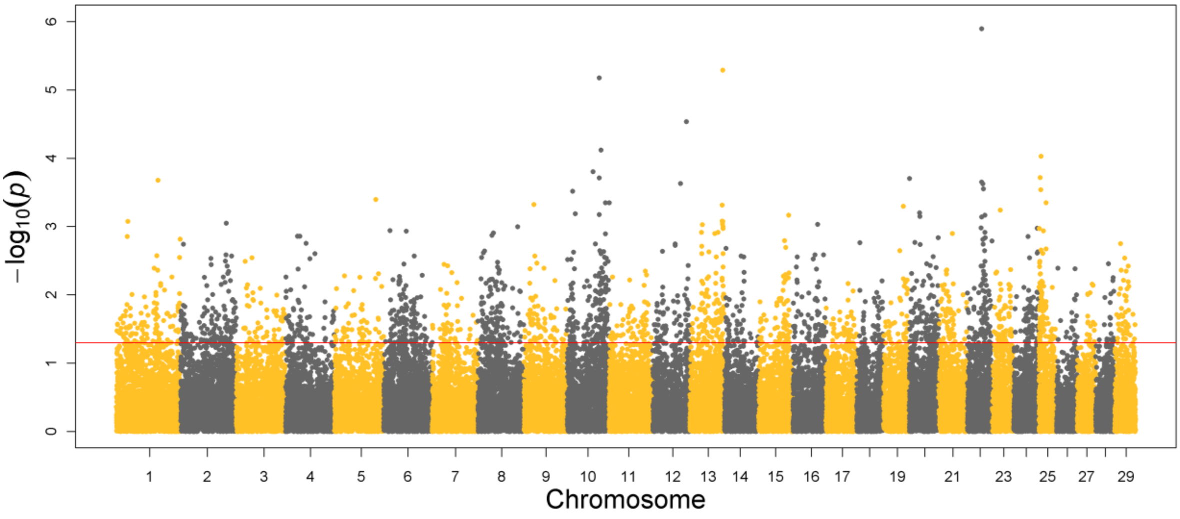

$-\log_{10}(p)$
0 1 2 3 4 5 6
1 2 3 4 5 6 7 8 9 10 11 12 13 14 15 16 17 19 21 23 25 27 29
Chromosome

(H) C8:1

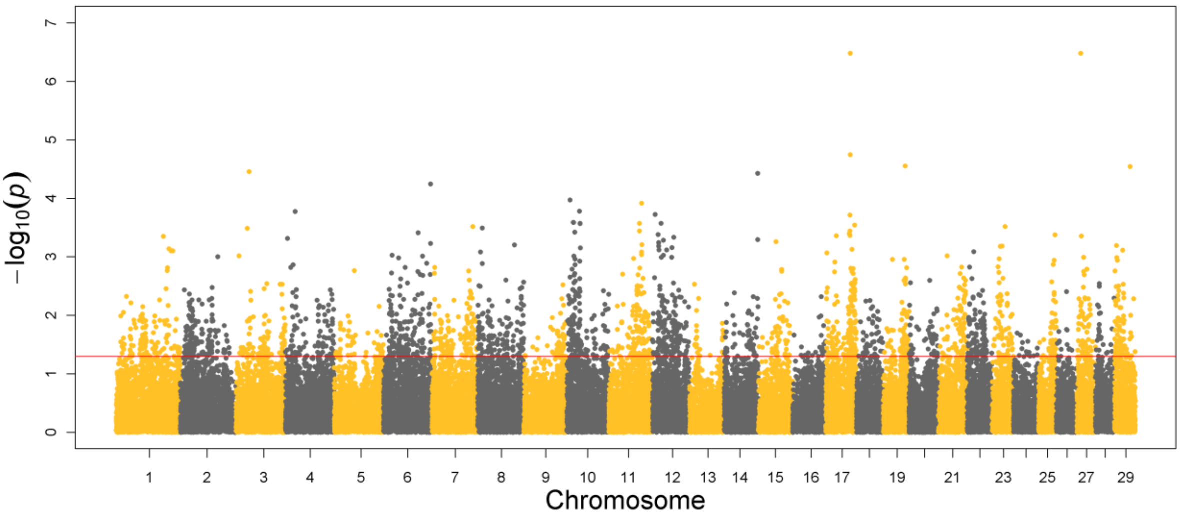

$-\log_{10}(p)$
0 1 2 3 4 5 6 7
1 2 3 4 5 6 7 8 9 10 11 12 13 14 15 16 17 19 21 23 25 27 29
Chromosome

(I) C14

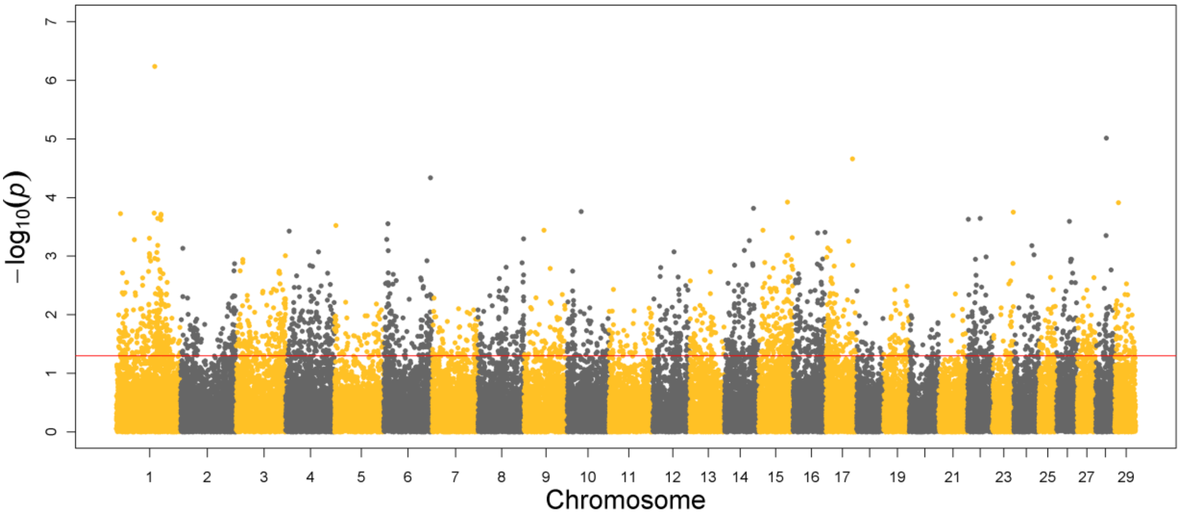

(J) C18

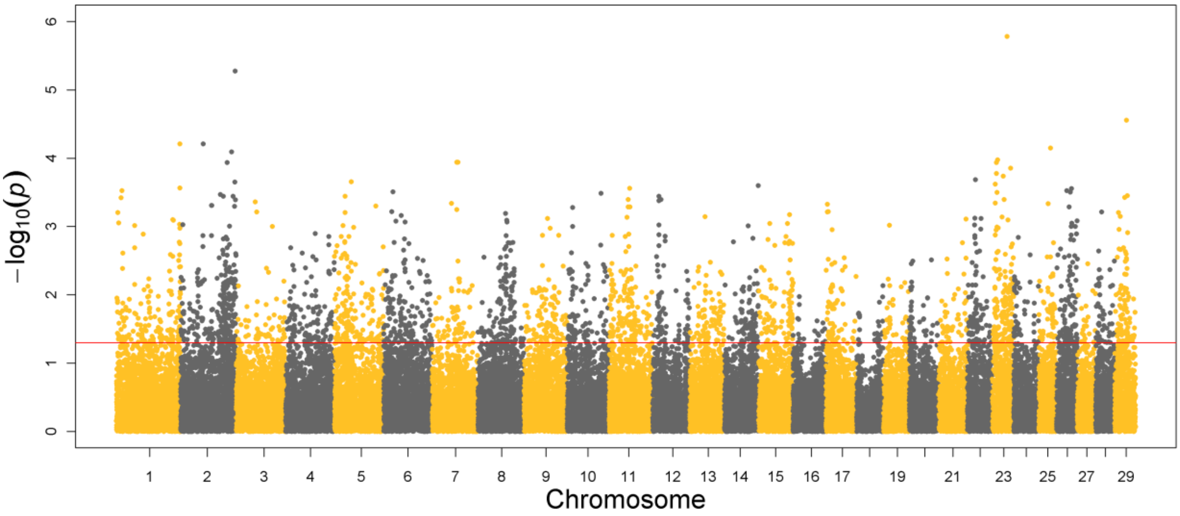

(K) PC_aa_C32:0

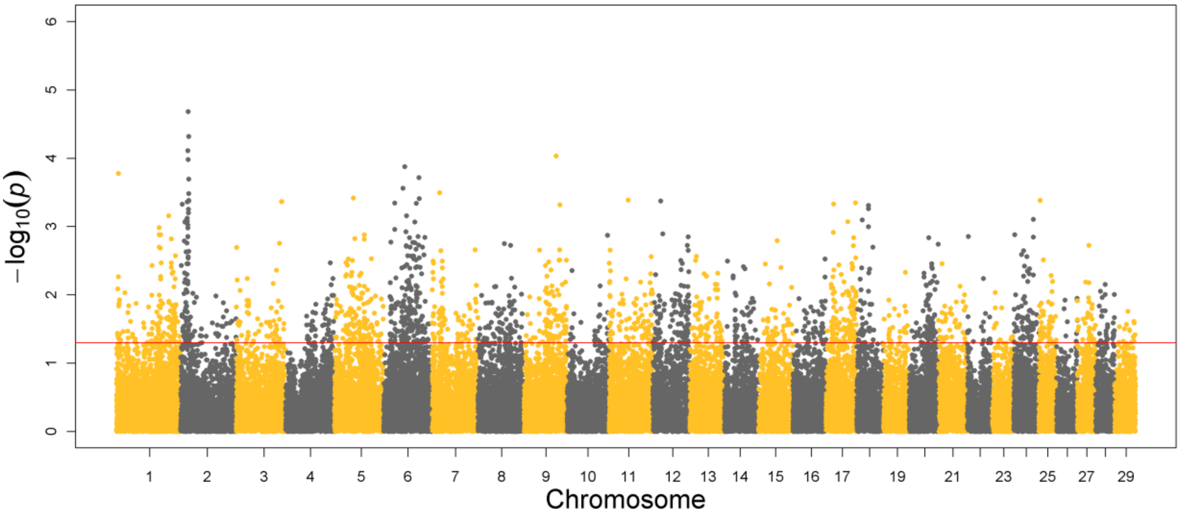

(L) PC_ae_C36:1

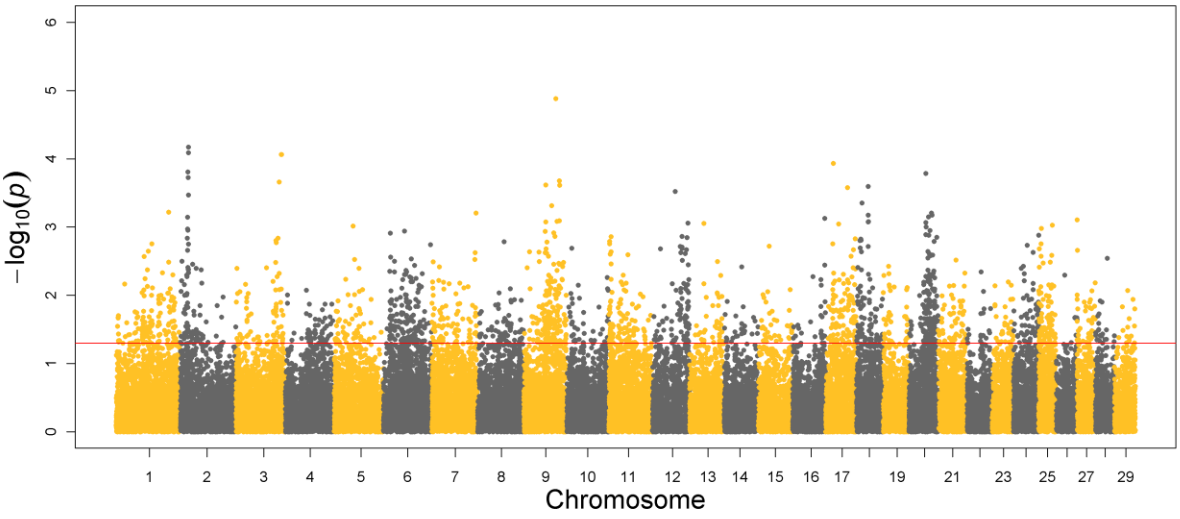

(M) SM_C20:2

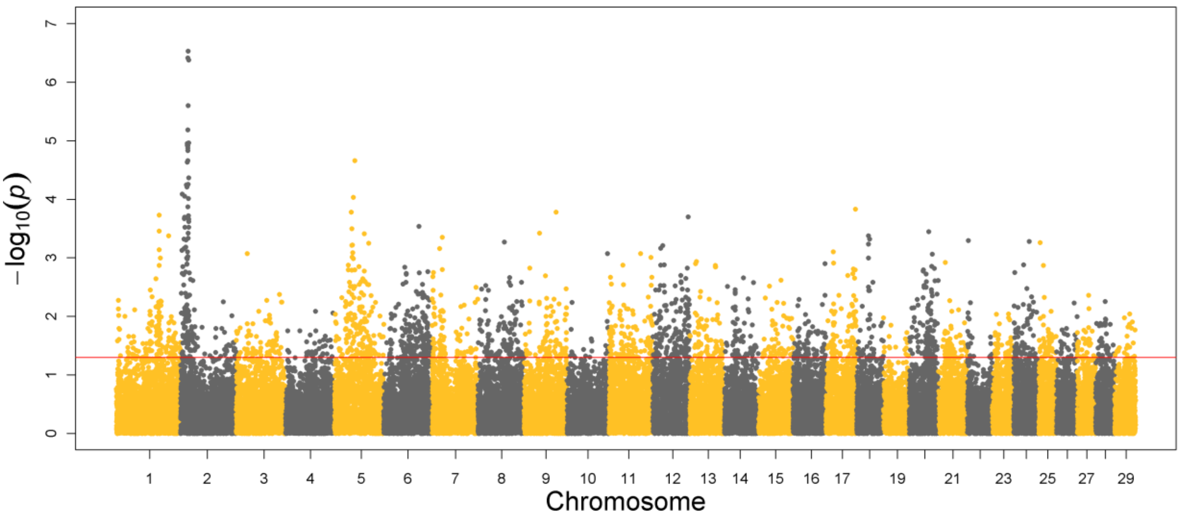

Supplement: Additional file 1 — Manhattan plots of GWAS results. Significance of association (−log10(p)) between SNPs across the bovine genome and the target traits from the groups of body weights (A,B), amino acids (C,D), acylcarnitines (E-J), phosphatidylcholines (K,L) and sphingomyelins (M) (N = 144 – 147). (A) total weight at month 9 (tw273), (B) daily weight gain from month 6 to 9 (dwg273), (C) arginine, (D) lysine, (E) free carnitine (C0), (F) acetylcarnitine (C2), (G) valerylcarnitine (C5), (H) suberylcarnitine (C8:1), (I) myristylcarnitine (C14), (J) stearoylcarnitine (C18) (K), diacylphosphatidylcholine C32:0 (PC_aa_C32:0), (L) acylethylphoshatidylcholine C36:1 (PC_ae_C36:1), (M) sphingomyelin C20:2 (SM_C20:2). [file 1471-2164-14-798-S1.pdf]
