# Supplementary material for: A systems biology approach using metabolomic data reveals genes and pathways interacting to modulate divergent growth in cattle
Source: BMC Genomics. 2013 Nov 18;14:798. doi: 10.1186/1471-2164-14-798 (PMC3840609; doi:10.1186/1471-2164-14-798)
Supplement: Additional file 4 — Components of the Gonadotropin releasing-hormone pathway that are encoded by genes from the growth network. [file 1471-2164-14-798-S4.pdf]

Additional_file_4: Components of the Gonadotropin releasing-hormone pathway that are encoded by genes from the growth network.

Full component name *	Abbreviation *	Corresponding gene in the growth network
Adenylate cyclase 1	AC	ADCY5
Calcium/calmodulin-dependent protein kinase II, alpha	CaMK	CAMK2G
Guanine nucleotide binding protein, alpha 11	Gq/11	GNAQ
Growth factor receptor-bound protein 2	Grb2	GRB2
Matrix metallopeptidase 2	MMP2	MMP2
Mitogen-activated protein kinase 6	MKK3/6	MAP2K6
Mitogen-activated protein kinase 10	JNK	MAPK10
Phospholipase A2	PLA2	PLA2G2, PLA2G5, PLA2G12B
Phospholipase C, beta	PLCβ	PLCB1, PLCB4
Protein kinase, cAMP-dependent, catalytic, delta	PKA	PRKACB
Protein kinase C	PKC	PRKCB, PRKCD
Protein tyrosine kinase 2, beta	Pyk2	PTK2B

* In accordance with the Kyoto Encyclopedia of Genes and Genomes (KEGG) nomenclature
§ Abbreviations correspond to the abbreviations in Figure 5
